# Fu Brick Tea as a Staple Food Supplement Attenuates High Fat Diet Induced Obesity in Mice

**DOI:** 10.3390/foods12244488

**Published:** 2023-12-15

**Authors:** Daying Wu, Haoan Zhao, Lei Guo, Xiukun Liu, Yan Liang, Qian Liu, Wei Cao, Xueyan Chen, Xin Gao

**Affiliations:** 1 Crop Research Institute, Shandong Academy of Agricultural Sciences/National Engineering Research Center of Wheat and Maize/National Key Laboratory of Wheat Breeding, Ministry of Science and Technology/Key Laboratory of Wheat Biology and Genetic Improvement in North Yellow & Huai River Valley, Ministry of Agriculture/Shandong Provincial Technology Innovation Center for Wheat, Jinan 250100, China; wudaying2020@163.com (D.W.); leiguo@nwsuaf.edu.cn (L.G.); liuxk0224@163.com (X.L.); 2 College of Food Science and Technology, Northwest University, Xi’an 710069, China; zhaohaoan@nwu.edu.cn (H.Z.); lianyan5517@126.com (Y.L.); liuqian2017@nwu.edu.cn (Q.L.); caowei@nwu.edu.cn (W.C.); 3 Institute of Crop Germplasm Resources, Shandong Academy of Agricultural Sciences, Jinan 250100, China

**Keywords:** Fu brick tea noodles, obesity, hyperlipidemia, inflammation, gut microbiota

## Abstract

Fu brick tea (FBT), a product of microbial fermentation from primary dark tea, also known as raw material tea (RMT), has been extensively studied for its functional properties. However, its potential as a staple food supplement for weight loss remains poorly understood. This study compared the weight loss effects of orlistat, traditional plain noodles (NN), and noodles supplemented with varying amounts of RMT (RMTN) and FBT (FBTN), with the aim to elucidate their lipid-reducing effects and underlying mechanisms. Experimental trials on high fat diet fed mice revealed significant weight loss, lipid-lowering, and hypoglycemic effects upon supplementation with orlistat, RMTN, and FBTN. Moreover, supplementation with orlistat, RMTN, and FBTN effectively restored serum and liver-related index levels, mitigating high-fat diet-induced dyslipidemia. Additionally, these supplements ameliorated liver and kidney damage by inhibiting oxidative stress and inflammatory responses. Furthermore, orlistat, RMTN, and FBTN exert their anti-obesity effects primarily by modulating genes associated with lipid metabolism and inflammatory responses and through regulation of the composition and structure of the gut microbiota. Importantly, FBTN demonstrated a significantly stronger lipid-lowering effect compared to RMTN, particularly at higher tea addition ratios. In contrast, NN supplementation exhibited minimal to no weight loss effects. Based on these findings, it could be inferred that FBT holds promise as a staple food supplement to ameliorate high-fat diet-induced obesity and its associated health conditions.

## 1. Introduction

Obesity, stemming from prolonged excessive long-term energy intake and inadequate physical activity [1], not only affects body size and quality of life but also predisposes individuals to various health risks such as type 2 diabetes, insulin resistance, cardiovascular disease, inflammation, and cancer. A prevalent symptom of this is fatty liver disease (hepatic steatosis), which, if left unaddressed, can progress to severe conditions like cirrhosis, fibrosis, and even liver cancer [2]. Additionally, obesity can impair kidney function, as indicated by decreased levels of serum creatinine, uric acid, and blood urea nitrogen [3]. Currently, common approaches for managing obesity include bariatric surgery and medications like orlistat and liraglutide. However, medication use may lead to undesirable side effects such as nausea, diarrhea, and loss of appetite [4]. As such, interventions based on exercise and dietary modifications remain the most effective and preferred strategies for alleviating obesity-related symptoms.

Tea, a popular non-alcoholic beverage enjoyed worldwide, has become a cherished part of daily life for people of all backgrounds. Based on flavanol oxidation, tea is classified into six categories: green tea, white tea, yellow tea, oolong tea, black tea, and dark tea [5]. Among these, dark tea stands out as a post-fermented tea, possessing unique sensory properties developed through microbial processes [6]. In China, major variants of dark tea include Anhua dark tea, Sichuan Tibetan tea, Hubei green brick tea, Guangxi Liubao tea, Yunnan Pu’er tea, and Shaanxi Fu brick tea. Fu brick tea (FBT), also known as Fu tea, appeared in Jing Yang, Shaanxi Province, during the Northern Song Dynasty in China. Initially available in loose form, tea merchants later transformed its packaging to address transportation challenges, leading to the creation of the first brick type of Fu tea around the first year of Ming Hongwu (1368 AD). Notably, Fu brick tea is distinctive due to its dominant bacteria, *Eurotium cristatum* [7]. An intriguing observation lies in the border areas of China, where ethnic groups consume diets rich in cholesterol and fat from meat and milk, yet exhibit lower incidences of hyperlipidemia compared to mainland China. This is attributed to their long-term consumption of FBT. Beyond its health benefits, Fu brick tea is widely loved for its mellow taste, and has gained popularity along the Silk Road in Central and West Asia.

FBT is primarily derived from primary dark tea, using raw material tea (RMT) as its basis. The production process involves a series of steps including piling, steaming, pressing, fermentation, and drying. These processes trigger a complex interplay of microbial community and metabolite changes within the RMT. Central to this transformation is the dominant bacterium, *Eurotium cristatum*, which plays a pivotal role. Firstly, it breaks down macromolecules such as starch, amino acids, and cellulose. Secondly, it causes the oxidation and condensation of polyphenols, forming compounds such as thearubigins and theabrownins. As a result of these reactions, the rough astringent flavor inherent in RMT diminishes, and the resulting tea broth exhibits a brighter, redder hue. Importantly, this transformation also enhances the anti-obesity, hypoglycemic, and other nutritional effects of FBT [8]. In recent decades, an array of studies has unveiled the multifaceted biological functions of FBT, particularly its potential in preventing obesity and reducing blood glucose levels by regulating the composition and structure of gut microflora [9]. FBT has also been found to curtail weight gain in mice by promoting the browning of subcutaneous fat, driving increased energy expenditure [10]. Additionally, interventions featuring FBT polysaccharides, polyphenols, and theaflavins have yielded promising outcomes in modulating gut microbiota, effectively reducing HFD-induced obesity, dysglycemia, oxidative stress, and inflammatory responses in mice [11,12,13]. Notably, *Eurotium cristatum* present in FBT increases the population of acetate- and butyrate-producing bacteria in the intestine of mice, thereby helping prevent fat accumulation and dyslipidemia [14]. Although previous reports have investigated the effects of FBT aqueous extract or its active components on HFD-induced hyperlipidemia in mice, comparative analyses between FBT and its raw material tea (RMT) have been limited to chemical composition and flavor assessments. Additionally, the potential of FBT used as a staple food has largely not been reported thus far. With an increasing emphasis on dietary health and concerns surrounding the high carbohydrate and fat content of traditional flour products, which pose risks of malnutrition, diabetes, and obesity [15], the demand for alternatives is on the rise. Furthermore, as tea alone might not suffice as a complete dietary solution, incorporating FBT into wheat flour to create noodles as a staple food presents an opportunity to enrich polyphenols, amino acids, protein, dietary fiber, and polysaccharides in the noodles. This enhancement endows these noodles with antioxidant, anti-inflammatory, and anti-obesity properties.

The objective of this study was to unravel the potential of FBT used as a staple food supplement. Our experiments utilized plain conventional noodles, RMT-supplemented noodles, and FBT-noodles to administer to mice, which were subjected to a high-fat diet. Our primary focus was to assess the anti-obesity effects of these noodle variations by examining the changes in weight, fat, blood lipids, oxidative stress, inflammation markers, and gene expression associated with obesity regulation in mice. Additionally, we delved into the regulatory effects of these noodle interventions on gut microbiota. Our findings suggest that FBT, when used as a staple food, demonstrates superior efficacy compared to RMT in preventing and treating diet-induced hyperlipidemia and obesity in mice.

## 2. Materials and Methods

### 2.1. Preparation of Different Tea Noodles

Raw material tea (RMT) and Fu brick tea (FBT) were supplied by Xianyang Jingwei Fu Tea Co., Ltd. (Xianyang, China), with a tea polyphenol content of 15.28% and 11.50%, and a tea polysaccharide content of 3.32% and 4.46%, respectively. The wheat variety Jimai 60 (JM) with medium gluten strength was obtained from Shandong Academy of Agricultural Sciences (Jinan, China). Wheat grains, RMT, and FBT were ground into a fine powder using a Brabender Quadrumat Senior (Brabender Instruments, South Hackensack, NJ, USA) and passed through a 100-mesh sieve. Normal noodle, designated as NN, was prepared using 100% JM wheat flour. Four different types of tea-supplemented noodles were created by replacing wheat flour with 6% RMT, 27% RMT, 6% FBT, and 27% FBT, designated as RMTN-6, RMTN-27, FBTN-6, and FBTN-27, respectively. The moisture content in each reconstituted dough ranged from 68.9% to 70.8%, determined through a mixing properties test on the Mixolab (Chopin Technologies, Tripette and Renaud, Paris, France). Subsequently, all types of cooked noodles were vacuum freeze-dried for 48 h, ground into powder, and stored at −20 °C for subsequent analyses.

### 2.2. Determination of the Nutritional Components of Noodles

The nutritional components of all noodle samples were analyzed following standard protocols outlined by the Association of Official Analytical Chemists (AOAC) [16]. Specifically, protein content was measured using the Kjeldahl method (AOAC Method No. 920.87), while crude fat content was assessed following the Soxhlet extraction method (AOAC Method No. 920.85). Carbohydrate content was measured using the enzymatic method (AOAC Method No. 2020.07). Dietary fiber content was calculated using the enzymatic–gravimetric method (AOAC Method No. 985.29). Crude ash content was evaluated through the combustion method (AOAC Method No. 923.03). All of the above values were used to calculate the total energy of each noodle sample.

### 2.3. Animals and Experimental Design

A total of 80 specific-pathogen-free C57BL/6J male mice, aged 5 weeks, with a body weight of 20 ± 2 g, were procured from the Beijing HFK Biotechnology Co., Ltd. (Beijing, China, Certificate No. SCXK<Jing>2019-0008). The mice were housed in a laboratory environment at an ambient temperature of 23 ± 2 °C and relative humidity of 55 ± 5%. They were subjected to a 12/12 h cycle and provided ad libitum access to standard chow and water. After a week of acclimation, all mice were randomly divided to a normal-diet group (ND, *n* = 10, 10% calories from fat) and a high-fat-diet group (HFD, *n* = 70, 45% calories from fat), with the latter used to induce obesity. After 10 weeks, the ND group continued to have access to a normal diet and distilled water. The 70 obese mice were further randomly subdivided into seven groups (10 animals each) based on body weight. Each subgroup received a different feeding regimen as follows: high-fat-diet (HFD group) plus distilled water, high-fat-diet plus orlistat as positive control (PC group, 54 mg kg^−1^ day^−1^), high-fat diet plus NN (NN group, 2400 mg kg^−1^ day^−1^), high-fat-diet plus RMTN-6 (RMTN-6 group, 2400 mg kg^−1^ day^−1^), high-fat-diet plus RMTN-27 (RMTN-27 group, 2400 mg kg^−1^ day^−1^), high-fat-diet plus FBTN-6 (FBTN-6 group, 2400 mg kg^−1^ day^−1^), and high-fat-diet plus FBTN-27 (FBTN-27 group, 2400 mg kg^−1^ day^−1^). Every day, an equal quantity of each of the five types of noodle powder was weighed into sterile and dry centrifuge tubes. Subsequently, a certain amount of water was added to create 200 mg/mL suspensions for each of the five types of noodles, each clearly labeled. The administration of distilled water and different samples was performed via gavage at 0.012 mL g^−1^. The diets were provided by Wuxi Fanbo Biotechnology Co., Ltd. (Wuxi, China), and their composition was described in Supplementary Materials Table S1. Each mouse was weighed weekly, and each food intake was measured daily throughout the intervention trial period. After 8 weeks, the mice were euthanized through anesthesia after 12 h fasting, followed by cervical dislocation. Blood samples were collected from the eyeballs of each mouse and centrifuged at 3000 rpm for 10 min at 4 °C. The serum samples were obtained from the resulting supernatant and then stored at −80 °C for further analysis. The samples of liver, kidney, and epididymal fat tissue were collected, rinsed with normal saline, weighed, snap-frozen in liquid nitrogen, and stored in a −80 °C refrigerator. A portion of the tissue was preserved and later fixed in 4% paraformaldehyde buffer for 48 h for subsequent histological analysis. Colonic content samples were collected for gut microbiota analysis and stored −80 °C until subsequent analyses.

The experiment protocol involving animals received approval from the Animal Ethics Committee at the Laboratory Animal Center of Northwest University (Approval ID: NWU-AWC-20220503M). All procedures adhered to the guidelines outlined in the National Institutes of Health for the Care and Use of Experimental Animals.

### 2.4. Oral Glucose Tolerance Test (OGTT)

Following 7 weeks of interventional treatment, mice were subjected to a 6 h fasting period before undergoing an oral glucose tolerance test (OGTT). The mice received 2.5 g/kg D-glucose via oral gavage, and blood samples were taken from their tail veins at 0, 30, 60, 90, and 120 min. Blood glucose levels were determined using a glucometer (Sinocare Biosensing Co., Changsha, China). The area under the curve (AUC) was computed using GraphPad Prism Software (Ver. 8.0.1, GraphPad Software Inc., San Diego, CA, USA) to quantify the OGTT result.

### 2.5. Histological and Immunohistochemical Examination

The liver, kidney, and epididymal fat tissue samples, fixed in 4% paraformaldehyde for 48 h, were embedded in paraffin, sliced into 5 μm-thick slices, and subsequently stained with hematoxylin and eosin (H&E). An optical microscope (TE2000-U, Nikon, Tokyo, Japan) was employed for histopathological examination to assess steatosis, inflammation, and hepatocyte ballooning following established methods [17]. The quantification of the epididymal fat area was performed using the Image J program (Ver. 1.53t, National Institutes of Health, Bethesda, MD, USA).

In addition, liver sections of mice were subjected to immunohistochemical analysis of the nuclear factor-κ-gene binding (NF-κB) p65, following previously described protocols [18]. Staining involved the NF-κB p65 monoclonal antibody (Ym3111, 1:200, Servicebio, Wuhan, China) as the primary antibody and goat anti-mouse (GB23301, 1:200, Servicebio, Wuhan, China) as the secondary antibody. An optical microscope (TE2000-U, Nikon, Tokyo, Japan) was used to capture images.

### 2.6. Biochemical Analyses of the Serum and Liver Samples

Parameters of serum lipid content, encompassing total cholesterol (TC), total triglycerides (TG), low-density-lipoprotein cholesterol (LDL-C), and high-density-lipoprotein cholesterol (HDL-C), were determined adhering to the manufacturer’s instructions. Additionally, the activities of liver enzymes, including serum aspartate transaminase (AST) and alanine aminotransferase (ALT), and the activities of oxidative-stress-related enzymes such as serum superoxide dismutase (SOD) and glutathione (GSH) were assessed in accordance with the manufacturer’s protocols. Moreover, renal function indicators including serum creatinine (CRE), uric acid (UA), and blood urea nitrogen (BUN) were analyzed. All the kits used were procured from Jiancheng Bioengineering Institute, Nanjing, China.

For liver tissue analysis, each portion of liver tissue (200 mg each) from mice in different groups was homogenized in 1.8 mL physiological saline in an ice water bath. The homogenate was centrifuged at 3500× *g* rpm at 4 °C for 15 min to produce supernatant for subsequent biochemical analysis. Concentrations of TC, TG, LDL-C, HDL-C, SOD, GSH, malonaldehyde (MDA), and nitric oxide (NO) in the liver were determined using the methods described in the corresponding commercial kits (Jiancheng Bioengineering Institute, Nanjing, China). Levels of liver inflammatory cytokines, such as tumor necrosis factor-alpha (TNF-α), interleukin-1beta (IL-1β), and interleukin-6 (IL-6) were quantified using mouse-specific commercial ELISA kits from Servicebio, Wuhan, China.

### 2.7. RNA Extraction and Real-Time Quantitative PCR

Liver tissue total RNA was extracted using TRIzol reagent (Servicebio, Wuhan, China), and RNA concentration and purity were measured with Nanodrop 2000 spectrophotometer (Thermo Fisher Scientific, Waltham, MA, USA). Subsequently, mRNA was reverse-transcribed into cDNA using a First Strand cDNA Synthesis Kit (Servicebio, Wuhan, China), and the expression levels of the related genes in the liver samples were determined by RT-qPCR. The primer sequences used for PCR in the experiments were provided in Supplementary Materials Table S2. GAPDH (glyceraldehyde 3-phosphate dehydrogenase) served as an internal reference, and the obtained signals and data were processed and analyzed following the 2^−ΔΔCT^ method.

### 2.8. Analysis of the Gut Microbiota by 16S rRNA Sequencing

Bacterial 16S rRNA gene sequence analysis and statistical assessments were conducted on mouse colon contents preserved at −80 °C from different groups. Bacterial genomic DNA was extracted using the OMEGA Soil DNA Kit (Omega Bio-Tek, Norcross, GA, USA), followed by the evaluation of DNA molecule size using 0.8% agarose gel electrophoresis. DNA quantification was carried out with a NanoDrop NC2000 spectrophotometer (Thermo Fisher Scientific, Waltham, MA, USA). The amplification of DNA utilized the forward primer 338F (5′-ACTCCTACGGGAGGCAGCA-3′) and the reverse primer 806R (5′-GGACTACHVGGGTWTCTAAT-3′), targeting the highly variable V3–V4 region of the bacterial 16S rRNA gene. Quantification of PCR amplicons was performed with the Quant-iT PicoGreen dsDNA Assay Kit (Invitrogen, Carlsbad, CA, USA). The purified amplified fragments were pooled in equal amounts and then double-end sequenced at 2 × 250 bp on the Illumina NovaSeq platform (Illumina, San Diego, CA, USA).

The sequence data underwent primary analysis and processing using QIIME2 and the R packages (Ver. 3.2.0). Alpha diversity indices such as the Chao1 richness estimator and Shannon diversity index were calculated through QIIME2. Structural alterations in microbial communities across different samples were investigated using beta diversity analysis visualized through principal coordinate analysis (PCoA) and nonmetric multidimensional scaling (NMDS). For additional comparison of species composition differences between samples, species composition analyses were performed using heat maps. Clustering outcomes were calculated for each sample and group using R software (Ver. 3.2.0), and heat maps were constructed using abundance data for the top 20 genera in terms of mean abundance.

### 2.9. Statistical Analysis

The experimental data underwent analysis employing independent one-way ANOVA, followed by the least significant difference (LSD) post hoc test using SPSS software (Ver. 26.0, SPSS Inc., Chicago, IL, USA) and GraphPad Prism software (Ver. 8.0.1, GraphPad Software Inc., San Diego, CA, USA). The values were expressed as the means ± standard deviation (*p*-value < 0.05).

## 3. Results and Discussion

### 3.1. The Supplement of FBT Changed the Nutritional Composition of Noodles

The morphological appearance of NN and noodles supplemented with RMT and FBT is depicted in Figure S1. Compared with the NN group, the RMTN-6 and FBTN-6 exhibited a glossy brown color with better stripe formation, whereas the RMTN-27 and FBTN-27 showed a darker brown color with poorer stripe formation, particularly noticeable in the RMTN-27. The darker color of the tea-added noodles is attributable to the high content of thearubigins and theabrownins in tea. Additionally, we have provided the nutritional composition data for each group of noodles in Table 1. The addition of RMT and FBT increased the protein content and decreased the fat content of the noodles. Specifically, a 6% tea addition increased the carbohydrate content and decreased the dietary fiber content of the noodles. Surprisingly, a 27% tea addition substantially decreased the carbohydrate content and increased the dietary fiber content. In addition, a 27% tea addition increased the ash content of the noodles. These changes in the nutritional composition also affected the overall energy content of the noodles. As FBT and RMT are richer in protein, dietary fiber, tea polysaccharides, and minerals and lower in fat [19], a higher percentage of tea addition could potentially improve the overall nutritional profile of the noodles.

### 3.2. Impact of FBTN on Fat Accumulation, Serum Biochemical Indices, and OGTT in HFD-Induced Mice

**Figure 1:**
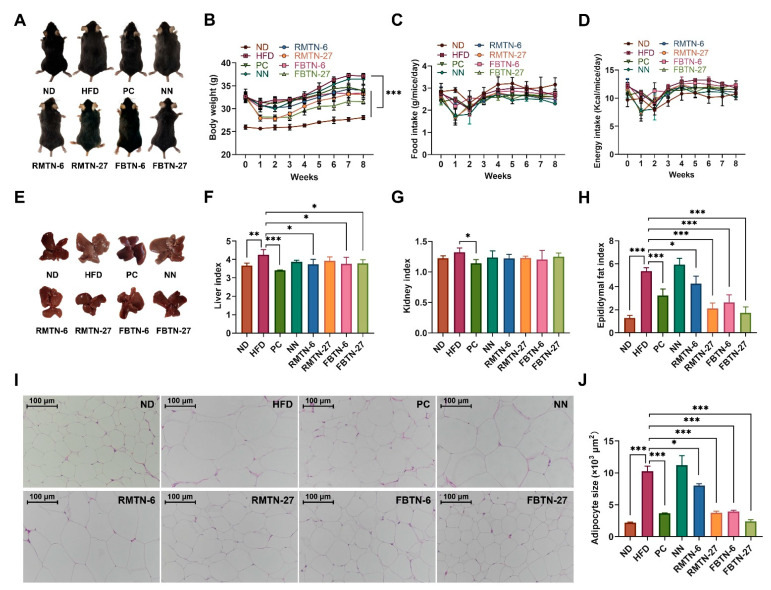
FBTN reduced body weight and fat accumulation in HFD-induced mice. (**A**) Representative photographs, (**B**) body weight, (**C**) food intake, (**D**) energy intake, (**E**) representative photographs of liver tissue, (**F**) liver index, (**G**) kidney index, (**H**) epididymal fat index, (**I**) representative H&E staining images of epididymal fat (200×), scale bar: 100 μm and (**J**) adipocyte size. Values are presented as the mean ± SD. Definitions of symbols: (*) *p* < 0.05, (**) *p* < 0.01, and (***) *p* < 0.001 compared to the HFD group.

Before initiating the intervention experiment, the body weights did not show significant differences among the HFD-fed groups but they were significantly higher than those in ND-fed mice (Figure 1B, week 0), indicating the successful establishment of the mouse obesity model. Toward the end of the experiment, mice in the HFD group exhibited significantly higher weights than those in the ND group, although their weights did not differ significantly from those in the NN group. Notably, supplementation with orlistat or with both RMTN and FBTN significantly inhibited weight gain, with the most marked effect observed in the FBTN-27 group (Figure 1A,B). There were no significant differences in food intake and energy intake between the HFD group and the six treatment groups (Figure 1C,D), indicating that weight loss was not associated with energy intake. A similar experiment found no reduction in mean energy intake in HFD-induced mice with *Ganoderma lucidum* intervention [20]. The livers of mice in the HFD and NN groups showed an abnormal grayish-white color, whereas the livers of mice in the PC, 6%, and 27% RMTN and FBTN groups displayed a normal reddish-brown color, similar to that of the ND group mice livers. Organ indices directly reflect the lipid content in organs. Oral administration of orlistat, 6%, and 27% RMTN and RBTN suspensions significantly suppressed HFD-induced elevations in the liver index and epididymal fat index, but did not affect the kidney index, except for the PC group (Figure 1F–H). These results suggest that supplementation with RMTN and FBTN leads to a reduction in visceral lipid deposition and may have a similar effect on diet-induced liver steatosis to orlistat.

Histological examination of the epididymal fat clearly revealed more significant adipocyte hypertrophy in the HFD group than in the ND group, while NN supplementation had no effect on this condition (Figure 1I,J). The increased adipocyte area is mainly the result of fat accumulation, leading to excessive obesity and the development of insulin-resistance and inflammation [21]. Interestingly, this increase in adipocyte size was significantly reversed in the orlistat and four tea noodle treatment groups, especially in the FBTN-27 supplemented mice, where the adipocytes returned to nearly the same size as those in the ND group mice. Studies have shown that orlistat has an inhibitory effect on lipase; thus, it is speculated that the inhibition effect of the RMTN and FBTN groups on the increase in epididymal fat cells in this study may be similar to orlistat [4]. Furthermore, it is evident that FBTN supplementation was more effective than RMTN supplementation in reducing adipocyte size. This observation aligns with a prior study that claims tea can reduce adipocyte size, with FBT demonstrating a greater efficacy [22,23].

**Figure 2:**
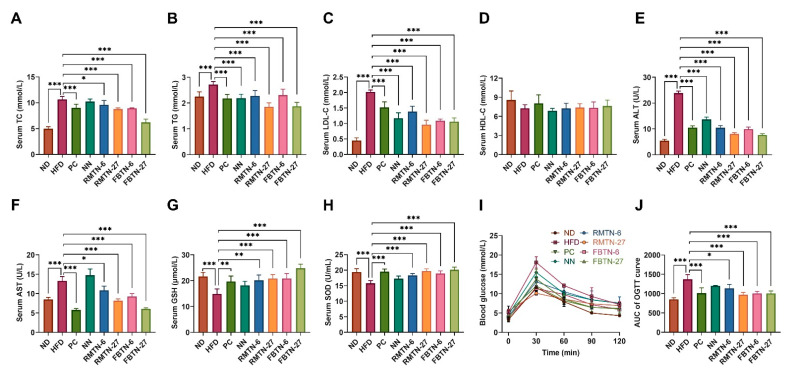
FBTN improved the serum biochemical indices and OGTT in HFD-induced mice. Serum (**A**) TC, (**B**) TG, (**C**) LDL-C, and (**D**) HDL-C levels; serum (**E**) ALT, (**F**) AST, (**G**) GSH, and (**H**) SOD levels; (**I**) oral glucose tolerance test (OGTT), and (**J**) AUC of OGTT curve. Values are presented as the mean ± SD. Definitions of symbols: (*) *p* < 0.05, (**) *p* < 0.01, and (***) *p* < 0.001 compared to the HFD group.

Obese individuals typically develop severe hyperlipidemia, a condition that can be assessed by measuring lipid profiles or serum lipid levels of TC, TG, LDL-C, and HDL-C. Serum-based lipid panels and lipoproteins data obtained from mice are presented in Figure 2A–D. Serum TC, TG, and LDL-C levels were significantly elevated in the HFD group in comparison to the ND group but the HDL-C levels did not show any marked difference. In this study, NN supplementation did not significantly affect serum TC levels, but orlistat and RMTN and FBTN supplementation effectively decreased the levels of TC, TG, and LDL-C induced by HFD. It is worth noting that tea polyphenols have previously been shown to reduce body weight and lipid metabolism levels in HFD-induced mice [24]. These results suggest that the interventions with FBTN and RMTN could achieve a similar effect to orlistat in ameliorating the abnormalities of lipid metabolism in obese mice, potentially due to the rich content of tea polyphenols. ALT and AST are key indicators closely related to liver injury, with elevated serum levels indicative of liver cell damage [25]. GSH and SOD are important anti-oxidative stress indicators and their levels are used to predict the health status of mice and the development of hyperlipidemia [26]. As shown in Figure 2E–H, the mice in the HFD and NN groups showed significantly higher serum ALT and AST levels but significantly lower GSH and SOD levels, compared to the mice in the ND group. These levels returned to normal after treatment with orlistat, 6%, or 27% RMTN and FBTN. Notably, the results indicated that FBTN supplementation was more effective than RMTN supplementation in alleviating the dyslipidemia, repairing liver injury and relieving oxidative stress than NN treatment. FBT polysaccharides have been found to attenuate metabolic syndrome in mice by regulating lipid metabolism and expression of genes related to oxidative stress [27]. These results may be attributed to the higher content of Fu brick tea polysaccharides in FBTN.

Obesity can contribute to weaker glucose tolerance to some extent. An oral glucose tolerance test (OGTT) was conducted on each group of mice one week prior to the conclusion of the experiment. The results showed that the fasting blood glucose levels in the ND group and the other six intervention groups were lower than those in the HFD group (Figure 2I). Additionally, the area under the curve of OGTT for HFD and NN groups was notably elevated, which was reversed after orlistat, 6%, or 27% RMTN and FBTN treatment (Figure 2J). The test demonstrated that both RMTN and FBTN can decrease blood glucose levels in mice, with FBTN showing a greater efficacy than RMTN under low-dose intervention. One of the key pathways in glucose metabolism involves glycogen synthesis. FBT has been shown to influence glycogen synthesis by modulating the expression of specific proteins and mRNAs such as PI3K, AKT, and GSK3 [28]. Therefore, it is plausible that the mechanism through which RMTN and FBTN lower blood glucose levels involves the regulation of genes associated with glycogen metabolism.

### 3.3. Effect of FBTN on HFD-Induced Organ Damage in Mice

**Figure 3:**
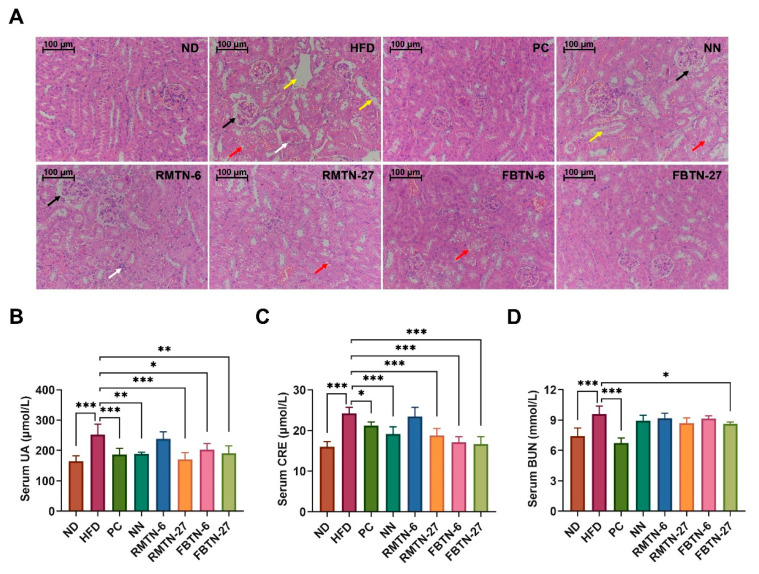
FBTN alleviated kidney damage in HFD-induced mice. (**A**) Representative H&E staining images of kidneys (200×), scale bar: 100 μm, serum (**B**) UA, (**C**) CRE, and (**D**) BUN levels. The arrows in (**A**) represent the glomeruli which were hypertrophic with some cell necrosis and thickened structural margins (black arrows): the tubules were swollen, with necrosis of epithelial cells and detachment into the lumen (yellow arrows), vacuolization (red arrows), and interstitial inflammatory cell infiltration (white arrows). Values are presented as the mean ± SD. Definitions of symbols: (*) *p* < 0.05, (**) *p* < 0.01, and (***) *p* < 0.001 compared to the HFD group.

Elevated glucose and adipose levels have been associated with detrimental effects on the structure and function of the kidney [29]. As depicted in Figure 3A, the kidney tissue in the control group displayed a clear tubular structure, normal glomerular size, well-defined structural margins without thickening, and no inflammatory cell infiltration. However, in the HFD and NN groups, the glomeruli exhibited hypertrophy with some cell necrosis and thickened structural margins (shown by black arrows), while the tubules appeared swollen, with necrosis of epithelial cells and detachment into the lumen (indicated by yellow arrows), vacuolization (indicated by red arrows), and a small amount of interstitial inflammatory cell infiltration (indicated by white arrows). Interestingly, RMTN-6 supplementation did not significantly alleviate the kidney damage. In contrast, the glomeruli of mice in the PC, RMTN-27, FBTN-6, and FBTN-27 groups displayed normal size and structure, with slight tubular dilatation and a small amount of epithelial cell vacuolation. Particularly, mice in the PC and FBTN-27 groups exhibited a similar degree of restoration in the kidneys compared to mice in the ND group. These results were further corroborated by the levels of kidney function biomarkers (Figure 3B–D), where serum UA, CRE, and BUN levels were all clearly reduced to varying degrees following treatments, particularly with orlistat and FBTN-27. Our study demonstrated that HFD induced kidney damage, as evidenced by the presence of glomerular hypertrophy, renal fibrosis, lipid droplet accumulation, and increased serum UAE in mice, which align with the observations made by Han et al. [30]. The protective effect on the kidney is attributable to the high polyphenol content in tea [31]. The different capabilities of FBTN and RMTN might come from the various types and levels of polyphenols, but the specific mechanism needs further study.

**Figure 4:**
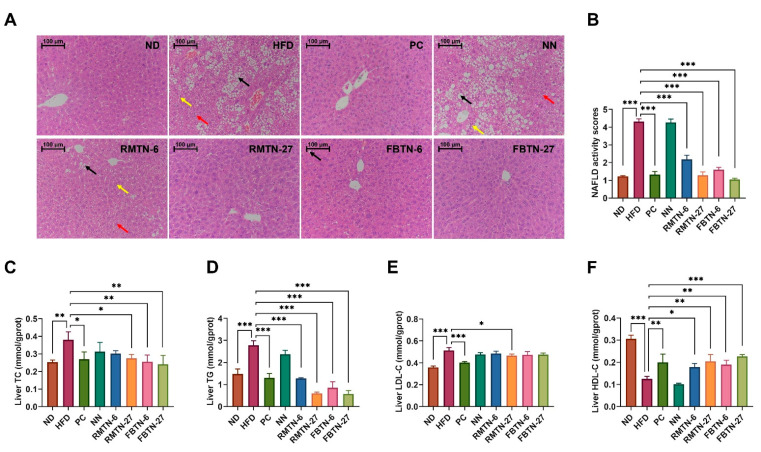
FBTN ameliorated the liver histopathological changes and lipid metabolism disorders in HFD-induced mice. (**A**) Representative H&E staining images of livers (200×), scale bar: 100 μm, (**B**) NAFLD activity scores for each group based on H&E sections, liver (**C**) TC, (**D**) TG, (**E**) LDL-C, and (**F**) HDL-C levels. The arrows in (**A**) represent lipid droplets (black arrows), cell swelling (red arrows) and inflammatory infiltrates (yellow arrows). Values are presented as the mean ± SD. Definitions of symbols: (*) *p* < 0.05, (**) *p* < 0.01, and (***) *p* < 0.001 compared to the HFD group.

**Figure 5:**
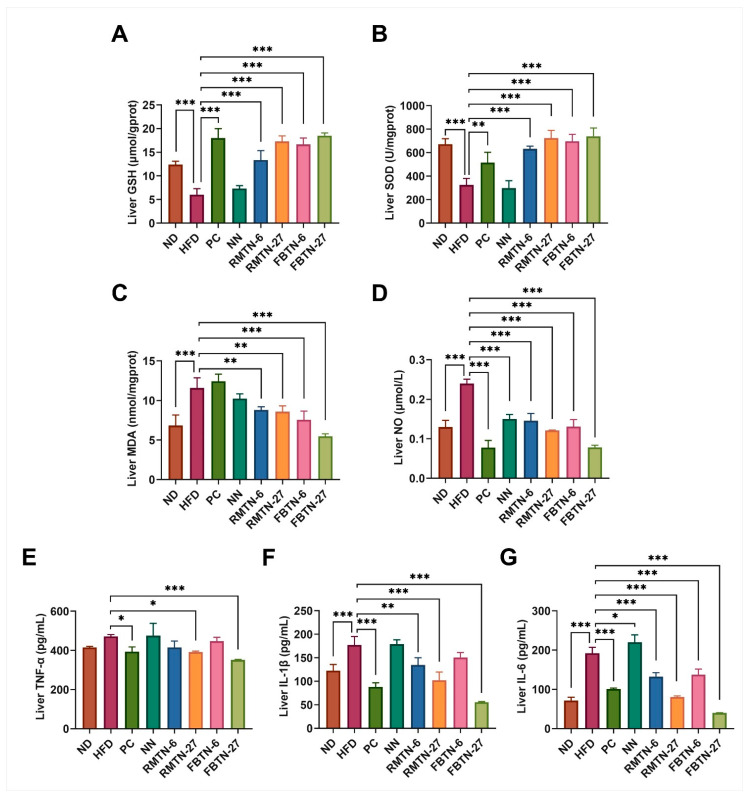
FBTN ameliorated the liver oxidative stress and inflammatory disorder in HFD-induced mice. Liver (**A**) GSH, (**B**) SOD, (**C**) MDA, and (**D**) NO levels; liver (**E**) TNF-α, (**F**) IL-1β, and (**G**) IL-6 levels. Values are presented as the mean ± SD. Definitions of symbols: (*) *p* < 0.05, (**) *p* < 0.01, and (***) *p* < 0.001 compared to the HFD group.

Figure 4A presents the histopathological morphological images of the mouse livers in different groups. Liver tissue sections from mice in the ND group displayed no discernible pathological symptoms. However, in the HFD and NN groups, a significant number of lipid droplets were observed (indicated by black arrows), along with more pronounced swelling (indicated by red arrows) and mild inflammation (indicated by yellow arrows). The accumulation of lipid droplets in hepatocytes can disrupt liver structure and function, and reducing this accumulation can prevent liver injury [32]. Our results indicate that these symptoms were alleviated through intervention with orlistat and tea-added noodles. Blinded scoring revealed that the livers of mice in the HFD and NN groups displayed significantly higher non-alcoholic fatty liver disease (NAFLD) activity compared to the ND and other intervention groups, with the FBTN-27 group exhibiting the lowest scores (Figure 4B). The lower scores indicate substantial improvement in hepatic steatosis, liver tissue damage, and inflammation. Furthermore, intervention treatments involving orlistat, RMTN, and FBTN significantly reversed HFD-induced abnormalities in the livers, as confirmed by the TC, TG, HDL-C, and LDL-C levels, with FBTN-27 displaying particularly clear effects (Figure 4C–F). The results suggest that low-dose RMTN treatments are less effective in inducing weight loss compared to FBTN, while RMTN in high doses is comparable to FBTN. FBT is a fermented tea, and during fermentation, a substantial amount of tea polyphenols is converted to theabrownins [33], which are considered the primary active compounds in black tea responsible for weight loss [34]. Similar studies have also indicated that fermented Qingzhuan tea has a stronger effect on hyperlipidemic mice than semi-fermented Qingmao tea [35]. FBT contained significantly higher levels of theabrownins compared to RMT, which could explain why FBTN was more effective at promoting weight loss due to its higher theabrownins content compared to RMTN. A continuous high-fat diet disrupts the dynamic balance between oxidation and antioxidation in the organism, leading to the generation of oxidative stress. This, in turn, triggers inflammatory responses and various hepatic diseases in the body [36]. For the analysis of oxidative stress and antioxidant status, the MDA, GSH, SOD, and NO levels in the liver tissue were determined, as presented in Figure 5A–D. The HFD resulted in a significant decrease in GSH and SOD levels and a dramatic increase in MDA and NO levels in the mouse livers. However, supplementation with orlistat, RMTN, and FBTN was effective in reducing the oxidative stress levels by regulating the biomarkers, namely, MDA, GSH, SOD, and NO in the liver, which were previously affected by the HFD and NN.

Moreover, obesity frequently gives rise to systemic chronic inflammation [37]. As illustrated in Figure 5E–G, the HFD group exhibited higher IL-1β and IL-6 levels in the liver compared to the ND group. However, no significant differences were observed in TNF-α, IL-1β, and IL-6 levels compared to the NN group. Orlistat effectively mitigated these adverse effects induced by the HFD. As is expected, both RMTN and FBTN supplementation significantly reduced the levels of liver inflammatory cytokines. It is evident that orlistat, RMTN, and FBTN were all effective in suppressing the systemic inflammation in mice induced by the high-fat diet, with FBTN demonstrating superior efficacy compared to RMTN. The polyphenols in FBT significantly alleviated the inflammation in the livers of mice fed with a high-fat diet. This effect was achieved by decreasing the levels of pro-inflammatory cytokines such as TNF-α and IL-6 while simultaneously increasing the levels of the anti-inflammatory cytokine IL-10 [12]. Furthermore, findings from a separate study suggest that one mechanism through which exogenous interventions reduce body weight in obese mice is by decreasing pro-inflammatory cytokines [38]. Hence, the variation in the effect of the two tea noodles on pro-inflammatory factors partly accounts for the discrepancies in the reduction in body fat observed in mice.

### 3.4. Effect of FBTN on the Expression of Genes Related to Lipid Metabolism and Inflammatory Responses in the Liver of HFD-Induced Mice

**Figure 6:**
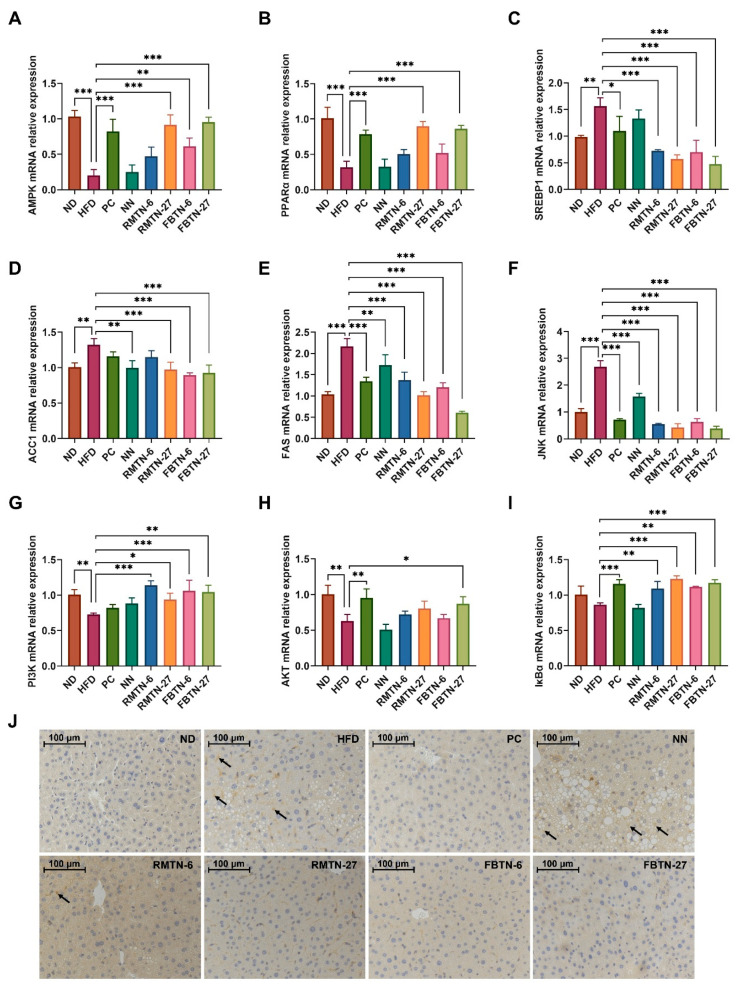
FBTN regulated the expression of genes related to lipid metabolism and inflammatory response in the livers in HFD-induced mice. (**A**–**I**) show the relative mRNA expression of liver AMPK, PPARα, SREBP1, ACC1, FAS, JNK, PI3K, AKT, and IκBα, and (**J**) NF-κB p65 immunohistochemical staining images of livers (200×), scale bar: 100 μm. Black arrows indicate positive cells. Values are presented as the mean ± SD. Definitions of symbols: (*) *p* < 0.05, (**) *p* < 0.01, and (***) *p* < 0.001 compared to the HFD group.

To delve deeper into the mechanisms underlying the effects of orlistat, RMTN, and FBTN interventions on lipid metabolism and inflammatory responses, we assessed the expression of related genes in the livers. In comparison to the ND group, the HFD group significantly decreased the expression levels of liver AMPK and PPARα genes while dramatically upregulating the expression of SREBP1, ACC1, and FAS genes (Figure 6A–E). However, NN supplementation only reduced the expression levels of ACC1 and FAS genes when compared to the HFD group. In contrast, orlistat, RMTN, and FBTN supplements significantly inhibited HFD-induced hepatic steatosis and reversed HFD-induced alterations in the gene expression of these genes, which caused inflammatory responses in the livers of the mice. AMPK plays a crucial role in various biological responses, including lipid synthesis and inflammatory regulation, making it essential for maintaining cellular functions [39]. PPAR-α is known to promote fatty acid oxidation and maintain lipid metabolic homeostasis in vivo by reducing the accumulation of lipid droplets in hepatocytes [40]. SREBP-1C acts as a key transcription factor regulating triglyceride biosynthesis and downstream ACC and FAS genes [41]. In our study, we postulated that orlistat, RMTN, and FBTN reduce the levels of lipids through two potential mechanisms. Firstly, these interventions may promote AMPK phosphorylation, leading to the regulation of SREBP-1c and subsequent downregulation of ACC and FAS expression, effectively inhibiting lipid synthesis [42]. Secondly, these interventions may upregulate lipid catabolism-related genes, such as PPAR-α, thus accelerating the oxidative breakdown of lipids [43].

Furthermore, inflammation-related regulatory genes were studied. The relative expression levels of liver JNK genes were significantly increased in the HFD group, but they returned to normal in the PC group and the groups including treatment with the five noodles (Figure 6F). While the HFD significantly reduced PI3K and AKT gene expression levels, it had no significant effect on IκBα gene expression compared to the ND group (Figure 6G–I). NN supplementation had no significant effect on PI3K, AKT, and IκBα gene expression in comparison to the HFD group. However, orlistat, RMTN, and FBTN each regulated the expression of inflammatory-factor-related genes to varying degrees, with FBTN supplements at higher doses showing more significant effects. Immunohistochemical analysis further demonstrated that the number of NF-κB p65 positive cells was significantly increased in the HFD and NN groups, whereas there were almost no positive cells after treatment with orlistat, RMTN-27, and FBTN (Figure 6J). JNK, a mitogen-activated protein kinase, is widely involved in inflammatory responses, cell proliferation, and apoptosis after targeted activation [44]. PI3K, a lipid kinase, leads to the activation of downstream AKT genes which regulate many processes, including metabolism, cell survival, and apoptosis, by phosphorylating downstream substrates [45]. In the inflammatory responses, activated AKT and JNK activate IκB kinases to promote the phosphorylation of IκB [46]. Normally, NF-κB is localized in cytoplasm in an inactivated manner. And when IκB proteasome is degraded, NF-κB is activated and translocated into the nucleus to induce the expression of inflammatory factors, i.e., TNF-α, IL-6, and IL-1β and other related genes [47]. Some studies have reported that tea polyphenols and theaflavins have significant anti-inflammatory effects [48,49]. In addition, *Eurotium cristatum* has a protective effect against oxidative stress and inflammatory damage in cigarette-smoke-induced mouse lungs via the MAPK/JNK signaling pathway [50]. *Platycodon grandiflorus* root extract attenuates the inflammatory responses in high-fat mice by reducing JNK phosphorylation and activating the PI3K/AKT pathway [51]. In our experiment, orlistat, RMTN, and FBTN supplementation mitigated the inflammatory responses in hyperlipidemic mice by downregulating JNK gene expression and upregulating PI3K, AKT, and IκBα gene expression, ultimately resulting in a decrease in downstream NF-κB protein and related inflammatory factor levels. Furthermore, research has indicated that Liubao brick tea can enhance glucose metabolism by activating the PI3K-Akt signaling pathway, thereby mitigating insulin resistance [52]. Therefore, it is reasonable to assume that orlistat, RMTN, and FBTN may lower blood glucose levels in mice by activating the PI3K-Akt pathway. These findings suggest that RMTN and FBTN stabilize lipid metabolism in obese mice by modulating genes associated with lipid synthesis and lipid metabolism, while also inhibit inflammatory responses in obese mice by regulating inflammation-related genes and proteins, thereby exhibiting similar anti-inflammatory effects as orlistat does.

### 3.5. Effect of FBTN on the Composition and Structure of the Gut Microbiota of HFD-Fed Mice

**Figure 7:**
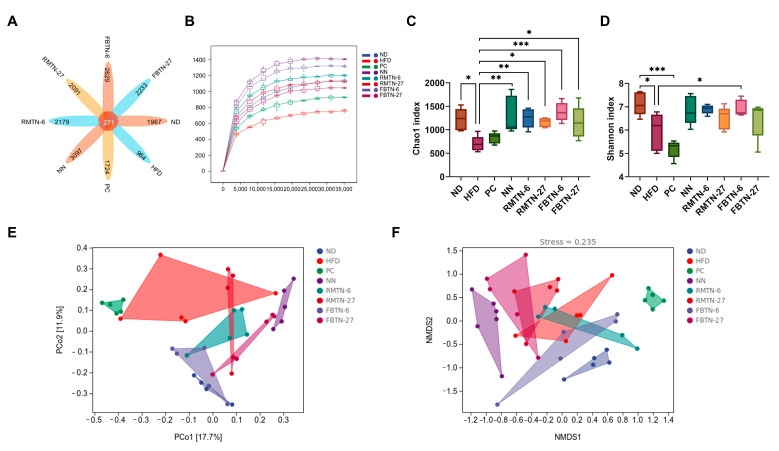
FBTN supplementation reverses gut microbiota dysbiosis in HFD-induced mice. (**A**) Venn plots showing OTUs in gut microbiota among groups, (**B**) rarefaction curve, (**C**) Chao1 index, (**D**) Shannon index, (**E**) Principal coordinate analysis (PCoA), and (**F**) Nonmetric multidimensional scaling (NMDS) of gut microbiota. Values are presented as the mean ± SD. Definitions of symbols: (*) *p* < 0.05, (**) *p* < 0.01, and (***) *p* < 0.001 compared to the ND and HFD group.

The intestinal microbiota are crucial to maintaining normal metabolism and the health of the body [53]. Obesity is closely linked to dysbiosis of the gut bacteria. Tea has been reported for its beneficial functions for weight loss and weight maintenance, attributed in part to its role in restoring disturbed intestinal microbiota. After quality filtering, denoising, splicing, and de-chimerization, a total of 3,352,965 high-quality reads were obtained from 48 samples. Operational taxonomic unit analysis was used to study associations among species of the gut bacteria. In total, 271 common OTUs were identified in the species across the eight groups; 1967, 964, 1724, 3097, 2179, 2091, 2629, and 2233 unique OTUs were found in the mice of the ND, HFD, PC, NN, RMTN-6, RMTN-27, FBTN-6, and FBTN-27 groups, respectively. These results indicate that intervention with noodle supplements led to higher species diversity in the intestinal microbiota of the mice (Figure 7A). The rarefaction curve illustrated the sequencing data covered a substantial portion of the diversity of the microbial community and captured almost all possible new phylotypes present in the samples (Figure 7B). Chao1 and Shannon indices are common parameters used to evaluate the alpha diversity of microbial species. These indices for the HFD group were significantly decreased compared to the ND group, demonstrating that HFD reduced the richness and diversity of the intestinal microbiota of mice (Figure 7C,D). In contrast, the data from the five noodle treatment groups, particularly from the FBTN-6 group, showed contrasting effects, in comparison to the HFD group. These results suggest that both types of noodle interventions, with or without tea supplements, increased the richness and diversity of intestinal microbiota in high fat diet fed mice. However, mice in the orlistat-treated group had the lowest Shannon index, likely due to the fact that orlistat induced fatty diarrhea in mice, resulting in lower concentrations of colonic contents [54]. Furthermore, beta diversity analysis is used to simplify the data structure by reducing the dimensionality of the multidimensional sample distance matrix. It characterizes the distance distributions within or between sample groups at a specific scale based on a chosen beta diversity metric [55]. Principal coordinate analysis and non-metric multidimensional scaling illustrate that the ND group and the HFD group were separated from each other, indicating that HFD caused significant changes in the composition of the gut microbiota. The gut microbiota composition in the mice of the PC group and the five noodle intervention groups differed notably from that of the HFD group. The orlistat and NN intervention groups displayed a significantly different gut microbiota composition than the ND group, while the RMTN and FBTN intervention groups, especially the FBTN-6 group, exhibited a highly similar gut microbiota composition to the ND group (Figure 7E,F). These results suggest FBTN-6 intervention was effective in restoring the gut bacterial flora disrupted by HFD. Intestinal flora dysbiosis, otherwise, leads to intestinal barrier impairment, and endotoxin lipopolysaccharide release triggers inflammatory response and obesity [20]. Fu brick tea has been reported to prevent obesity by restoring gut microbiota to healthy conditions [22]. As such, the effective RMTN and FBTN interventions can explain their anti-obesity effects. Furthermore, these results also indicate that generally FBTN had a stronger modulatory effect on intestinal flora than RMTN did. Cheng et al. reported similar results that fermented Qingzhuan tea was more effective than Qingmao tea in restoring diverse intestinal microbiota [35].

**Figure 8:**
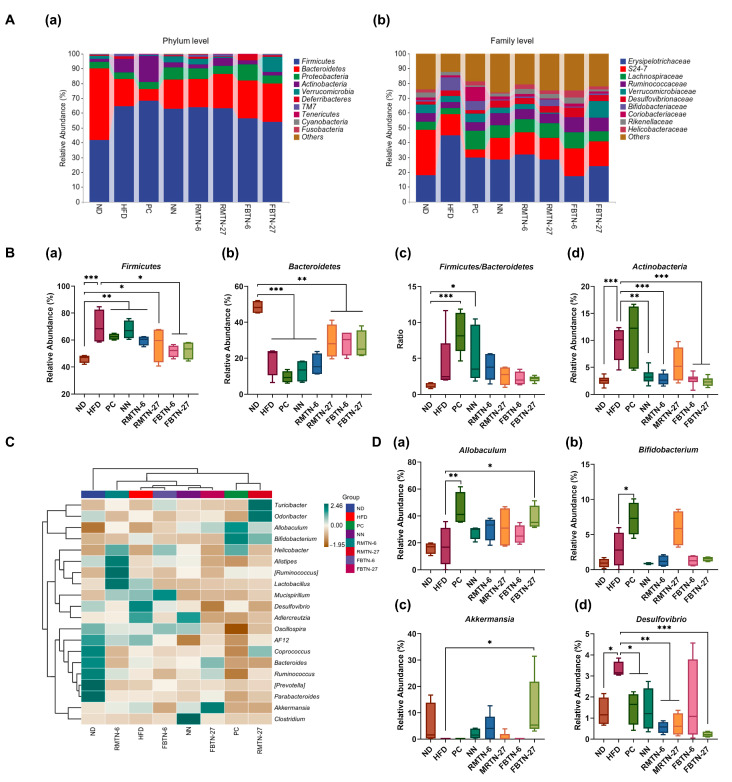
(**A**) Relative abundance at the (**a**) phylum and (**b**) family level, (**B**) differences in the abundances of gut microbial community at the phylum level between groups, including (**a**) Firmicutes, (**b**) Bacteroidetes, (**c**) the ratio of Firmicutes/Bacteroidetes, and (**d**) Actinobacteria, (**C**) heatmap of the top 20 gut microbiota at the genus level for each group, and (**D**) differences in the abundances of the gut microbial community at the genus level between groups, including (**a**) Allobaculum, (**b**) Bifidobacteria, (**c**) Akkermansia, and (**d**) Desulfovibrio. Values are presented as the mean ± SD. Definitions of symbols: (*) *p* < 0.05, (**) *p* < 0.01, and (***) *p* < 0.001 compared to the ND and HFD group.

The effects of the orlistat, RMTN, and FBTN interventions on gut microbiota at the phylum level in obese mice are shown in Figure 8A(a),B. The results highlight that the dominant phyla in the intestinal microbiota of all mice were *Firmicutes*, *Bacteroidetes*, *Proteobacteria,* and *Actinobacteria*. In the HFD group, the abundance of *Firmicutes* and *Actinobacteria* increased from 46.16% and 2.53% to 69.97% and 9.29%, respectively, and the abundance of *Bacteroidetes* decreased from 48.44% to 19.21%. Notably, RMTN and FBTN interventions restored the abundance of these bacteria to varying degrees, with FBTN being more effective. The increase in body fat in mice is often associated with an elevated Firmicutes/Bacteroidetes (F/B) ratio [56]. In the study, the F/B ratio tended to increase as a result of HFD feeding. But this tendency was partially reversed by supplementation with RMTN and FBTN, though it did not reach statistical significance. Similar studies have reported that FBT polysaccharide and *Artemisia sphaerocephala* Krasch polysaccharide did not counteract the HFD-induced increase in the F/B ratio [27,57]. Moreover, the F/B ratio was significantly higher in the NN group compared to the ND group, which may contribute to the increased body weight of the mice receiving NN supplementation. Surprisingly, orlistat intervention did not significantly alter the abundance of the aforementioned bacterial groups. This observation differs somewhat from the results of previous studies and may be due to drug specificity and the individuality of the animals [58]. Further, as seen in Figure 8A(a), FBTN-27 significantly increased the abundance of the probiotic *Verrucomicrobia*, a group associated with nutrient metabolism. This suggests that FBTN-27 could act as a beneficial diet for improving the intestinal flora.

At the family level, the more abundant microbiomes were *Erysipelotrichaceae*, *S24-7* and *Lachnospiraceae* (Figure 8A(b)). The data from this study demonstrate that the addition of orlistat and noodles, with or without tea, significantly reduced the relative abundance of *Erysipelotrichaceae* in the HFD-fed mice. Prior studies have indicated that *Erysipelotrichaceae* may promote the absorption of energy from food, leading to elevated blood lipids and weight gain in the host [59]. Moreover, a substantial enrichment of Erysipelotrichaceae can result in elevated levels of inflammatory factors including TNF, potentially worsening inflammation-related diseases in the host [60]. Conversely, supplementation with orlistat, NN, RMTN, and FBTN all resulted in increased *Lachnospiraceae* levels compared to the HFD-fed group. A decrease in the abundance of *Lachnospiraceae* can result in a reduction in the levels of short-chain fatty acids such as butyric acid, which in turn leads to lower HDL-C levels and increased blood lipids [61]. These findings suggest that RMTN and FBTN could regulate lipid metabolism and inflammatory responses by influencing specific gut microbiota.

The relative abundance of the 20 most abundant bacteria at the genus level was further investigated to assess the microbiota composition of colon contents from the eight test groups (Figure 8C,D). In comparison to the ND group, HFD increased the abundance of *Desulfovibrio*, *Mucispirillum*, *Adlercreutzia*, and *Lactobacillus*, while decreasing the abundance of *Ruminococcus*, *Bacteroides*, *Coprococcus*, *Alistipes*, *Odoribacter*, *Parabacteroides*, *Prevotella*, and *Oscillospira*. Notably, *Lactobacillus*, a recognized probiotic, was significantly enriched in the intestine of the HFD-fed mice, likely due to organismal differences in mice, in agreement with previous findings [58,62]. Furthermore, obesity triggers inflammatory responses in tissues or organs associated with the hosts’ systemic metabolic homeostasis, and the persistence of inflammation exacerbates fat accumulation [63]. Studies have shown that short-chain fatty acids play a role in weight loss by regulating host energy expenditure and inhibiting inflammation through the modulation of the expression of relevant cytokine genes [64]. However, HFD led to a reduction in butyric acid-producing bacteria such as *Ruminococcus*, *Odoribacter*, *Coprococcus*, and *Allobaculum*, which promote the oxidative breakdown of fatty acids and have beneficial effects on intestinal health by reducing inflammatory responses [65]. *Mucispirillum* is a bacterium closely associated with inflammation, and its proliferation in HFD-fed mice causes inflammation in the organism [66]. Similarly, *Desulfovibrio* causes intestinal inflammation as well as disruptions of the homeostasis of lipid and blood glucose via the secretion of endotoxins such as lipopolysaccharides [67]. Interestingly, the intervention of orlistat, RMTN, and FBTN resulted in a significant reduction in the abundance of *Desulfovibrio*, *Adlercreutzia,* and *Mucispirillum*. Furthermore, in comparison to the HFD group, the abundance of *Allobaculum* and *Bifidobacterium* was higher in the PC group, the abundance of *Clostridium* and *Oscillospira* was higher in the NN group, the abundance of *Alistipes* and *Lactobacillus* was higher in the RMTN-6 group, the abundance of *Coprococcus*, *Turcicactor*, *Odoribactor*, *Allobaculum*, and *Bifidobacterium* was higher in the RMTN-27 group, the abundance of *Mucispirium*, *Oscillospira*, and *Turcicactor* was higher in the FBTN-6 group, and the abundance of *Akkermansia*, *Bacteroides*, *Ruminococcus,* and *Allobaculum* was higher in the FBTN-27 group. *Akkermansia* belongs to *Verrucomicrobia* and degrades mucin to produce propionic acid [68]. Studies have shown that *Akkermansia* is crucial for the metabolic function of intestinal barrier and fighting inflammation, and that it regulates abnormal lipid metabolism by reducing TG levels and promoting white fat browning, facts that have a significant negative correlation with body weight [69]. *Bifidobacterium*, also a probiotic, breaks down polysaccharides and mucins, producing lactic acid and acetic acid, thereby inhibiting weight gain in mice [70]. *Bacteroides*, one of the important dominant bacteria, stimulates nutrient conversion and promotes the formation of propionic acid, which was significantly enriched in mice with reduced body weight after dietary intervention [71]. Conversely, an increase in *Clostridium* and *Oscillospira* is associated with detrimental effects on the body’s intestinal barrier, leading to severe oxidative stress and inflammation. This increase in abundance is positively correlated with weight gain [72]. This could explain why NN intervention did not prevent HFD-induced disorders of lipid metabolism in mice. Prior studies have shown that tea consumption can improve gut microbiota composition and structure in obese mice, restoring the abundance of *Akkermansia*, *Bacteroides*, *Allobaculum*, and *Bifidobacterium*, and thus contributing to weight loss [22,73,74]. In summary, the modulatory effects of orlistat, RMTN, and FBTN on HFD-induced disorders of lipid metabolism and inflammation in mice were attributed to increased levels of beneficial intestinal bacteria and decreased levels of harmful bacteria.

## 4. Conclusions

This study has revealed the potential of incorporating both RMT and FBT into plain noodles to enhance their nutritional profile and functionality. Orlistat, RMTN, and FBTN have demonstrated significant capabilities in reducing HFD-induced weight gain, body fat accumulation, and abnormal blood lipid levels in mice by activating the AMPK/ACC and PPARα pathways. Furthermore, these interventions effectively inhibited obesity-induced glucose accumulation and systemic inflammation through modulation of the PI3K/AKT pathway and JNK/NF-κB pathway. In addition, supplementation with RMTN and FBTN had a positive impact on the gut microbiota composition. Notably, FBTN was more effective in alleviating the obesity-related symptoms caused by HFD compared with RMTN. Taken together, this study underscores the potential of FBT as a natural and beneficial supplement when incorporated into staple foods. Beyond enhancing nutritional value, FBT shows promise in preventing and ameliorating diseases associated with obesity. These findings provide compelling evidence supporting the potential of FBT as an obesity-preventive supplement in staple foods.

## Figures and Tables

**Table 1:** The nutritional components analysis of noodles.

Nutritional Component	Sample				
	NN	RMTN-6	RMTN-27	FBTN-6	FBTN-27
Energy (KJ)	1403.04 ± 0.52	1494.36 ± 0.12 ***	1272.77 ± 0.43 ***	1408.42 ± 0.52	1281.98 ± 0.14 ***
Protein (g)	14.78 ± 0.13	15.66 ± 0.11 ***	17.63 ± 0.08 ***	15.37 ± 0.04 ***	17.12 ± 0.09 *
Fat (g)	1.31 ± 0.15	0.89 ± 0.21 ***	0.87 ± 0.01 ***	0.91 ± 0.02 ***	0.59 ± 0.01 ***
Carbohydrate (g)	61.89 ± 0.41	67.73 ± 0.35 ***	49.79 ± 0.29 ***	63.34 ± 0.46 ***	50.51 ± 0.37 ***
Dietary fiber (g)	8.48 ± 1.24	7.93 ± 0.57 ***	13.70 ± 0.01 ***	7.06 ± 0.81 ***	15.30 ± 1.05 ***
Ash (g)	1.01 ± 0.01	1.02 ± 0.00	2.01 ± 0.01 ***	1.10 ± 0.00 ***	2.11 ± 0.02 ***

Values were calculated on a dry basis of 100 g samples and expressed as the mean ± standard deviation (*n* = 3). Definitions of symbols: (*) *p* < 0.05 and (***) *p* < 0.001 compared to the NN group.

## Data Availability

The raw sequencing data are publicly available and accessible through the NCBI Sequence Read Archive (SRA) database (BioProject Number: PRJNA1008041).

## Supplementary Materials

The following supporting information can be downloaded at: https://www.mdpi.com/article/10.3390/foods12244488/s1, Figure S1: Appearance of the different formulated noodles; Table S1: Quantitative PCR primer sequences used in this study; Table S2: Diet compositions and nutritional ingredients of the experimental mice.
