# Supplementary material for: Fu Brick Tea as a Staple Food Supplement Attenuates High Fat Diet Induced Obesity in Mice
Source: Foods. 2023 Dec 15;12(24):4488. doi: 10.3390/foods12244488 (PMC10743230; doi:10.3390/foods12244488)
Supplement: Supplementary file 1 [file foods-12-04488-s001.zip › foods-2730949-supplementary.pdf]

Supplementary Materials

Table S1. Diet compositions and nutritional ingredients of the experimental mice.

Product code	Normal diet (ND)	High-fat diet (HFD)
**Energy composition (%)**		
**Protein**	24.7	20.0
**Fat**	11.9	45.0
**Carbohydrate**	63.4	35.0
**Energy (kcal/g diet)**	**3.405**	**4.415**
**Ingredient (g/100 g diet)**		
**Corn Starch**	28.0000	14.4200
**Wheat flour**	36.0000	18.5400
**Casein**	0	12.0000
**Bean pulp**	16.0000	8.2400
**Maltodextrin**	0	2.0000
**Fish meal**	5.0000	2.5750
**Soybean**	2.5000	1.2875
**Soybean oil**	2.2000	1.1330
**Yeast powder**	0.8000	0.4120
**Alfalfa meal**	0.5000	0.2575
**Bran**	3.0000	1.5450
**Compound premix**	6.000	4.5900
**Lard**	0	18.0000
**Saccharose**	0	15.0000
**Total**	100.0000	100.0000

Table S2. Quantitative PCR primer sequences used in this study.

Gene name	Forward primer (5′→3′)	Reverse primer (5′→3′)
PPAR-α	CACTACGGAGTTCACGCATGT	GTGACATCCCGACAGACAGGC
SREBP1	CCATCGACTACATCCGCTTCTT	CAGGTCCTTCAGTGATTTGCTTT
FAS	TGTCCTGCCTCTGGTGCTTG	GCAAAATGGGCCTCCTTGATAT
AMPK	AACCTGAGAACGTCCTGCTTGAT	CTTCCTGAAATGACTTCTGGTGC
ACC1	TTTGTTTGGTCGTGACTGCTCTG	AGGATGTTCAACCTGTAGCCGAG
IκB-α	CACTTGGTGACTTTGGGTGCT	GCTGTATCCGGGTACTTGGG
PI3K	AAACTCCGAGACACTGCTGATG	GCTGGTATTTGGACACTGGGTA
AKT	CTTCCTCCTCAAGAACGATGGC	TGTCTTCATCAGCTGGCATTGT
JNK	GCCATTTCAGAATCAGACCCAT	CCCGATGAATAATTCCAGCAGA
GAPDH	CCTCGTCCCGTAGACAAAATG	TGAGGTCAATGAAGGGGTCGT

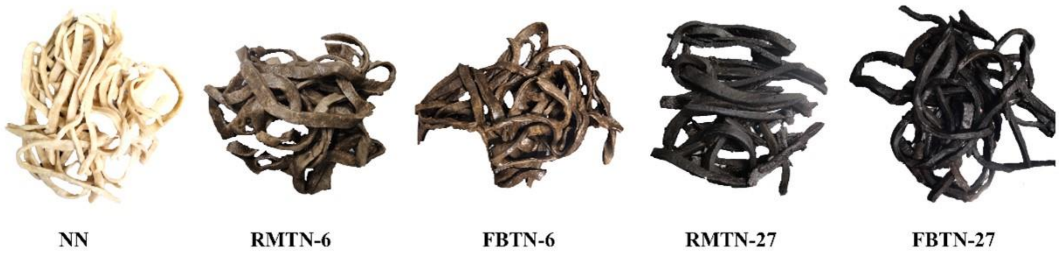

Figure S1. Appearance of the different formulated noodles.
